# The development of a novel curriculum to address past sexual assault

**DOI:** 10.5116/ijme.5643.a0d0

**Published:** 2015-11-21

**Authors:** Robin Mason, Janice Du Mont

**Affiliations:** 1 Women's College Hospital, Women's College Research Institute, Toronto, Ontario, Canada

**Keywords:** Curriculum development, past sexual assault

## Introduction

Sexual violence is experienced by women of every age, country, social class, culture and religion. In a recent American survey for example, an estimated 18% of women reported having been raped and an additional 45% experienced other forms of sexual violence at some point during their lifetime.^1^ The detrimental effects of sexual assault can be short-lived or persist for a lifetime. In addition to any immediate physical injuries sustained, sexually assaulted women can experience other negative sequelae such as HIV and other sexually transmitted infections; unwanted or intended pregnancies; psychological challenges such as anxiety, sleep disturbances, and attempted or completed suicide; difficulties in interpersonal relationships;as well as adverse life events including further future victimizations.^2^^-^^3^ Not surprisingly, women who have been sexually assaulted are more likely than those who have not to perceive their health as poorer and more frequently access health services.^2^ Thus, healthcare providers are often the first professional contact for survivors of sexual assault.^4^

While sexually assaulted women access the healthcare system, they do not necessarily disclose any information about the assault to their providers. In fact, a recent systematic review of disclosure of sexual assault to healthcare providers reported that across eight studies, just 6% to 27% of women ever disclosed to a health provider.^5^ In the one study to ask survivors to whom they first disclosed, 5% reported the first disclosure was to a physician.^6^

Disclosing sexual assault can be challenging due to a host of barriers including distrust of formal systems, concerns about confidentiality, and judgmental attitudes by healthcare providers which can exacerbate survivors’ feelings of embarrassment, self-blame, shame, and guilt.^7^^,^^8^ Yet not disclosing sexual assault has been associated with chronic negative mental health problems such as post-traumatic stress and depression.^9^ Most women eventually do tell someone about the sexual assault but fewer than half disclose within the first few days and approximately one third wait more than a year before disclosing.^10^^-^^13^

### From competencies to curriculum

Given the prevalence of sexual assault and the numerous associated negative health outcomes, it is clear that all health providers should have the requisite knowledge and skills to respond appropriately when a disclosure is made and to facilitate disclosure when indicators suggest the possibility of past sexual assault. Knowing how to do so is a matter of education and training and, in 2012, this was deemed a priority by the government of Ontario, Canada. In response, we undertook the development of an online curriculum suited to diverse health and allied healthcare providers. As the approach to health education is increasingly competency-based,developing core competencies to inform the curriculum was a necessary first step.^14^^-^^16^

In developing the competencies for our curriculum, we followed a method we had employed in developing other curricula.^17^ We started with a systematic literature search of OVID Medline, EMBASE, PsycInfo, and PubMed databases, limited to English language records published between 1985 and 2013 to determine what was known about disclosure of sexual assault to health providers.^6^ The findings clustered into two major categories, “Helpful Practices” and “Unhelpful Practices” and within these several minor themes were revealed. For example, “Helpful Practices” included: “Tangible Aid”, “Validating Disclosure”, and “Providing Emotional Support.” Within each of these, important behaviours were identified (e.g., “listening in an active or responsive way”, “telling the survivor she was not to blame”).

These findings were presented for discussion and review to members of an expert advisory committee formed to assist with the development of the curriculum. Members of the advisory committee had extensive expertise in the delivery of health care for victims of interpersonal violence and represented a range of disciplines and philosophical perspectives. After confirming the relevancy of our findings to clinical practice, the advisory committee provided further suggestions for providing competent care. For example, it was noted that “a stronger emphasis should be placed on providers examining their own values, beliefs, and experiences… as these often further victimize survivors”.

The agreed upon desirable behaviours, practices, and necessary knowledge were then rewritten in the form of competencies, sorted and aggregated following the updated version of Bloom’s taxonomy,^18^ and loosely organized as “know (factual knowledge), know oneself (self-reflection), know how (procedural knowledge)” items. Two overarching domains were identified: “Create an environment that supports disclosure” and “Respond appropriately to women who disclose”. Within each of these several specific behaviours were outlined, for example in the first domain were noted such behaviours as: “Recognise individual, relational, and societal barriers to disclosing sexual assault”; Create a physical environment that supports disclosure (e.g., use posters, pamphlets, ensure privacy)”, “Recognize how your own experiences, values, beliefs, and attitudes may influence your interactions with patients”.

Working with the competencies a framework for the curriculum incorporating the key elements in the provision of care to women who have experienced past sexual assault - whether or not they choose to disclose - was developed. The framework acknowledges the need to address misconceptions or myths about sexual assault and its victims before introducing new practice behaviours and simultaneously recognizes that despite the development of knowledge and understanding, the introduction of new practices may face challenges from within the work environment (e.g. lack of funding to produce pamphlets or posters that support disclosure). 

The next step was to prepare a series of scripts for interactive case scenarios and videos and submit these for review to subject matter experts in nursing, medicine, and occupational therapy. Working with a software development firm and a number of actors, the scripts were programmed, animated, uploaded, and piloted to determine whether they successfully engaged learners in achieving the competencies. Quizzes were incorporated into the online modules and a pre-test and post-test developed to track success in meeting the learning objectives and outcomes.

Our knowledge transfer strategy included bringing together the provincial leads of diverse health disciplines (e.g., medical technicians, midwives, physical therapists, occupational therapists, and nurses) to advise on how best to introduce the curriculum to their members. With their help, we developed postcards, advertisements, and presentations which were shared across the province.

The curriculum, *Addressing Past Sexual Assault in Clinical Settings, *was launched May 1, 2015.^19^ By Sept. 1, 2015 more than 1200 learners had completed the curriculum. Feedback from a very diverse group of health care professionals including nurses, therapists, radiation technologists, and addiction counsellors, has been extremely positive and generated comments such as; “just completed the excellent module”, “a great resource for all health care professionals” and “a great resource with lots of information that I will be using in my work with clients”. Future examination of pre- and post-test data will determine whether the curriculum has effected significant improvements in health providers’ knowledge, attitudes and skills when caring for women who may have experienced a past sexual assault. What is already clear is that this curriculum has addressed an identified gap in the education of health professionals and that by combining evidence gathered through the published literature with the tacit knowledge and expertise of frontline experts, the resulting curriculum is relevant, meaningful and useful to medical and other practitioners. This process of curriculum development can be readily applied to other topics and may improve learners’ engagement and achievement of educational outcomes.

### Conflict of Interest

The authors declare that they have no conflict of interest.
